# The lncRNA *Neat1* is required for corpus luteum formation and the establishment of pregnancy in a subpopulation of mice

**DOI:** 10.1242/dev.110544

**Published:** 2014-12

**Authors:** Shinichi Nakagawa, Masayuki Shimada, Kaori Yanaka, Mari Mito, Takashi Arai, Eiki Takahashi, Youko Fujita, Toshihiko Fujimori, Laura Standaert, Jean-Christophe Marine, Tetsuro Hirose

**Affiliations:** 1 RNA Biology Laboratory, RIKEN, 2-1 Hirosawa, Wako, Saitama 351-0198, Japan; 2 Laboratory of Reproductive Endocrinology, Graduate School of Biosphere Science, Hiroshima University, Higashi-Hiroshima 739-8528, Japan; 3 Support Unit for Animal Resources Development, Research Resources Center, RIKEN Brain Science Institute, 2-1 Hirosawa, Wako, Saitama 351-0198, Japan; 4 Women's Clinic Oizumi-Gakuen, Higashi-Oizumi, Tokyo 178-0063, Japan; 5 Division of Embryology, National Institute for Basic Biology (NIBB), Okazaki 444-8787, Japan; 6 Laboratory for Molecular Cancer Biology, Center for the Biology of Disease, VIB, Leuven 3000, Belgium; 7 Laboratory for Molecular Cancer Biology, Center of Human Genetics, KU Leuven, Leuven 3000, Belgium; 8 Institute for Genetic Medicine, Hokkaido University, Sapporo 060-0815, Japan

**Keywords:** *Neat1*, Paraspeckles, Corpus luteum, Progesterone, Sfpq, Stochastic failure

## Abstract

*Neat1* is a non-protein-coding RNA that serves as an architectural component of the nuclear bodies known as paraspeckles. Although cell-based studies indicate that *Neat1* is a crucial regulator of gene expression, its physiological relevance remains unclear. Here, we find that *Neat1* knockout (KO) mice stochastically fail to become pregnant despite normal ovulation. Unilateral transplantation of wild-type ovaries or the administration of progesterone partially rescued the phenotype, suggesting that corpus luteum dysfunction and concomitant low progesterone were the primary causes of the decreased fertility. In contrast to the faint expression observed in most of the adult tissues, *Neat1* was highly expressed in the corpus luteum, and the formation of luteal tissue was severely impaired in nearly half of the *Neat1* KO mice. These observations suggest that *Neat1* is essential for the formation of the corpus luteum and for the subsequent establishment of pregnancy under a suboptimal condition that has not yet been identified.

## INTRODUCTION

The nucleus is highly organized and divided into multiple nuclear compartments or nuclear bodies. These bodies contain specific sets of proteins and nucleic acids involved in particular nuclear processes (reviewed in Mao et al., 2011). Paraspeckles are one of the most recently identified nuclear bodies and contain a family of RNA-binding proteins called the DBHS (*Drosophila* behavior and human splicing) family proteins, which share common domain structures consisting of three RNA recognition motifs arranged in tandem (Fox et al., 2002; Bond and Fox, 2009). *Neat1* is a long noncoding (lnc) RNA that exclusively localizes to paraspeckles and serves as an architectural component of these nuclear bodies (Hutchinson et al., 2007; Clemson et al., 2009; Sasaki et al., 2009; Sunwoo et al., 2009; Chen and Carmichael, 2010). Both short (*Neat1*_*1*; 3 kb) and long (*Neat1_2*; 20 kb) isoforms of *Neat1* are transcribed from the *Neat1* locus using the same promoter; they are generated by alternative use of termination signals, the balance of which is regulated by the opposing actions of the CFIm complex and hnRNPK around the polyadenylation site of *Neat1*_*1* (Naganuma et al., 2012). The architectural function of each isoform is well characterized: *Neat1_2* plays an essential role in assembling paraspeckle components into the nuclear bodies, whereas *Neat1_1* cannot induce nuclear body formation by itself (Sasaki et al., 2009; Naganuma et al., 2012), although it can increase the number of paraspeckles when overexpressed in cells expressing *Neat1_2* (Clemson et al., 2009). Isoform-specific deletion of *Neat1_2* leads to the disappearance of paraspeckles, resulting in the even distribution of paraspeckle components throughout the nucleoplasm (Sasaki et al., 2009). In adult mice, *Neat1_1* is expressed in a wide range of tissues, whereas distinct *Neat1_2* expression is restricted to a limited population of cells (Nakagawa et al., 2011). Accordingly, prominent paraspeckle formation is observed only in a small population of cells expressing high levels of *Neat1_2* in living animals (Nakagawa et al., 2011). The limited formation of paraspeckles in animals is remarkably different from that of cultured cell lines that express both isoforms and form paraspeckles, except for embryonic stem cells (Chen and Carmichael, 2009). Therefore, paraspeckles are almost ubiquitous nuclear bodies *in vitro* but are cell population-specific nuclear bodies *in vivo*.

Two different mechanisms have been proposed for the molecular functions of paraspeckles. First, paraspeckles directly regulate the expression of adenosine-to-inosine hyper-edited mRNAs through the nuclear retention of these target transcripts (Prasanth et al., 2005; Chen et al., 2008; Chen and Carmichael, 2009). Second, paraspeckles indirectly regulate gene expression by serving as ‘molecular sponges’ that sequester and inhibit the function of paraspeckle-localizing components, such as Sfpq, that also function as transcriptional regulators (Hirose et al., 2014; Imamura et al., 2014). However, *Neat1* knockout (KO) mice, which lack paraspeckles, are viable and fertile (Nakagawa et al., 2011), leaving the physiological role of these nuclear structures unresolved.

To examine the physiological function of paraspeckles, we performed detailed phenotypic analyses of *Neat1* KO mice and found that nearly half of the naturally mated female mice stochastically failed to become pregnant. Serum progesterone levels are dramatically decreased in the affected mice, and the phenotype is considerably rescued by ovarian transplantation or by the administration of progesterone. We propose that *Neat1* assists in the establishment of pregnancy by stabilizing the formation of the corpus luteum under a set of unidentified suboptimal conditions.

## RESULTS

### Female *Neat1* KO mice stochastically fail to establish pregnancy

**Fig. 1.**
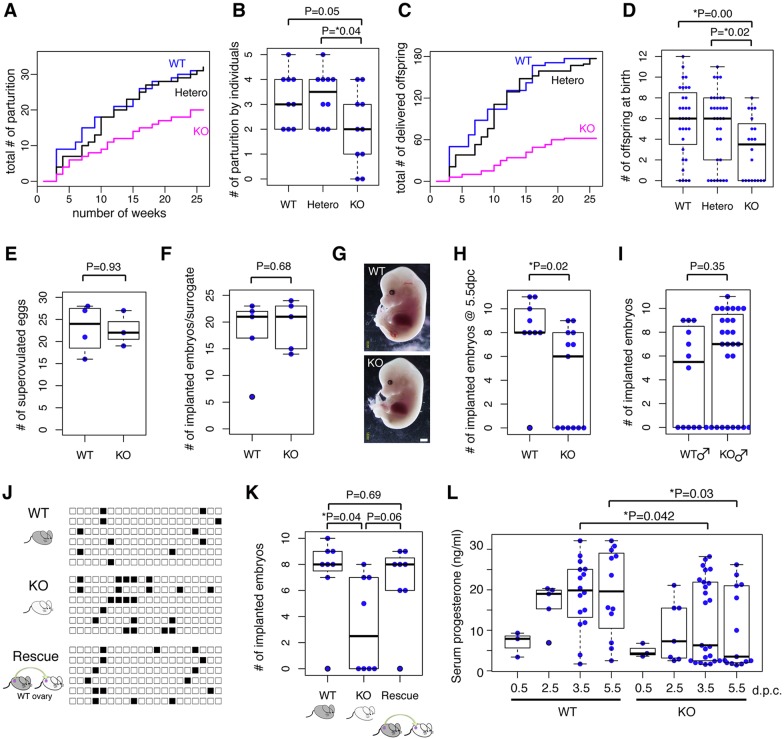
***Neat1* KO mice show decreased fertility.** (A) Plot of the cumulative number of parturition events for *Neat1* KO (magenta, *n*=10), heterozygous (black, *n*=10) and WT (blue, *n*=9) littermates. (B) Beeswarm boxplot of the number of parturition events by each individual. Each blue dot represents an individual mouse. (C) Plot of the cumulative number of pups delivered at parturition. (D) Beeswarm boxplot of the number of offspring delivered at parturition. Each blue dot represents an individual parturition event. (E) Beeswarm boxplot of the number of superovulated eggs. Each blue dot represents an individual mouse injected with hCG. (F) Beeswarm boxplot of the number of implanted embryos in a surrogate mother 14 days after transfer of *in vitro* fertilized eggs. The eggs were obtained from WT or *Neat1* KO mice and *in vitro* fertilized with sperm from C57BL/6 mice. A total of 30 fertilized eggs were transplanted. Each blue dot represents an individual surrogate. (G) External appearance of embryos developed from eggs obtained from WT or *Neat1* KO mice. Scale bar: 1 mm. (H) Beeswarm boxplot of the number of implanted embryos 5.5 days after natural mating. Each blue dot represents an individual. (I) Beeswarm boxplot of the number of implanted embryos recovered from plug-checked C57BL6 female mice mated with WT or *Neat1* KO male mice. The embryos were counted at 7.5-14.5 dpc. (J) Daily record of copulation of WT, *Neat1* KO and *Neat1* KO with transplanted ovary (Rescue) mice. Female mice were mated with vasoligated males, and copulation was checked by the formation of a plug in the morning. Each black box represents the day at which plug formation was observed. Each row represents an individual animal. (K) Beeswarm boxplot of the number of implanted embryos at 7.5-14.5 dpc in sham-operated WT, sham-operated *Neat1* KO and *Neat1* KO transplanted with WT ovary (Rescue) mice. Ovarian transplantation increased the number of successful pregnancies. Each blue dot represents an individual animal. (L) Beeswarm boxplot of serum progesterone levels. Approximately half of the *Neat1* KO mice failed to produce progesterone at high levels. Each blue dot represents an individual mouse. For all boxplots, the box represents the mean, 25th and 75th percentiles; whiskers show the maximum and minimum. All *P* values were calculated using a two-tailed, nonequal variance *t*-test.

During the course of maintaining the *Neat1* KO mouse colony, we noticed that only a small number of offspring could be obtained from *Neat1* KO females. To examine the fertility of the female *Neat1* KO mice in detail, KO (*n*=10), wild-type (WT; *n*=9) or heterozygous (*n*=10) littermates were mated with C57BL/6 male mice, and we counted the number of parturition events and the number of offspring born over 26 weeks. For all experiments mentioned below, the heterozygous parents had been extensively backcrossed to C57BL/6 to match the genetic background. The number of parturition events for the *Neat1* KO mice was notably decreased compared with that of the WT or heterozygous littermates (Fig. 1A,B). The decreased fertility was even more striking when we compared the number of offspring delivered at parturition (Fig. 1C,D). Notably, we observed that the same mice that had once delivered normally became stochastically infertile at the subsequent pregnancy, suggesting that the fertility of the *Neat1* KO mice was affected by certain environmental conditions rather than by the genetic-based polymorphism of the individual animals. To investigate the physiological mechanism underlying the decreased fertility of female *Neat1* KO mice, we analyzed the number of ovulated oocytes using a superovulation model. We could recover similar numbers of ovulated eggs from 3-week-old WT and *Neat1* KO female mice after the injection of human chorionic gonadotropin (hCG), suggesting that ovulation occurred normally (Fig. 1E). We could also obtain normal numbers of blastocysts from the uteruses of naturally mated *Neat1* KO mice at 3.5 days post coitum (dpc) (7±1, *n*=5; mean±s.d.), suggesting that eggs derived from the *Neat1* KO mice could undergo normal development. To further confirm this finding, we isolated eggs from WT or *Neat1* KO mice and transferred them to a pseudopregnant surrogate mother after *in vitro* fertilization. We could recover similar numbers of embryos with normal morphology at 14 days after the transfer (Fig. 1F,G), indicating that *Neat1* KO mice produced normal eggs. We then examined the number of implanted embryos in naturally mated female mice at 5.5 dpc, when implantation sites and embryos can be readily recognized by visual observation. We could not find any signs of implantation in 6 of 13 cases (Fig. 1H), suggesting that pregnancy was aborted around the time of implantation in these mice. However, we found no external abnormalities in the embryos and uteruses of the other seven *Neat1* KO mice. These results suggested that *Neat1* stochastically becomes indispensable for the establishment of pregnancy under certain conditions. By contrast, the number of embryos recovered from plug-checked C57BL6 female mice mated with male *Neat1* KO mice was not significantly different (*P*=0.35) compared with that recovered following mating with male Neat1 WT mice (Fig. 1I).

### The decreased fertility of *Neat1* KO mice is caused by ovarian defects

We then asked whether the lack of implantation was caused by defects in uterine function or by changes to the hormonal environment in the animals. To distinguish between these possibilities, WT and *Neat1* KO mice (six of each) were mated with vasoligated C57BL/6 males, and we examined whether pseudopregnancy, a process that occurs independently of a uterus or embryo, was induced in these animals. In WT females, pseudopregnancy was induced after the first copulation, and copulatory plug formation was not observed for more than 12 days in the same animal (Fig. 1J, WT). By contrast, *Neat1* KO mice frequently copulated at an interval of 3 or 4 days (Fig. 1J). We even observed successive plug formation for 2 to 4 days, which was never observed for WT mice (Fig. 1J). These results suggest that the *Neat1* KO mice fail to close the estrous period and that the subsequent induction of pseudopregnancy was impaired. Interestingly, this phenotype was partially rescued by the unilateral transplantation of WT ovaries into *Neat1* KO mice (Fig. 1J), suggesting that non-ovarian tissues were fairly normal in the *Neat1* KO mice. Unilateral ovarian transplantation also rescued, to some extent, the establishment of pregnancy after natural mating (*P*=0.06), and the number of implanted embryos was comparable to that of the WT littermates (Fig. 1K). Notably, we frequently observed embryos in the uterine horn, which was connected to the host mutant ovary in all cases, suggesting that ovulated eggs and embryos were normal in the *Neat1* KO mice, whereas the post-ovulatory ovary was responsible for the decreased fertility.

### Serum progesterone levels were decreased in *Neat1* KO mice

One of the most important functions of the post-ovulatory ovary is the generation of the corpus luteum and secretion of the steroid hormone progesterone, which is essential for the establishment and maintenance of pregnancy (reviewed in Stocco et al., 2007). We therefore examined serum progesterone levels in *Neat1* KO mice (Fig. 1L). In WT mice, serum progesterone levels were considerably increased during early pregnancy, whereas the level remained unchanged in a subpopulation of *Neat1* KO mice (Fig. 1L). At 3.5 dpc, 50% (9 of 18) of the *Neat1* KO mice failed to increase serum progesterone levels, and the average concentration was significantly lower compared with that of WT mice (18.8±8.6 ng/ml in WT and 12.5±10.3 ng/ml in KO, mean±s.d., *P*=0.042, Fig. 1L). The same trend was also observed at 5.5 dpc, when 53% (7 of 13) of the *Neat1* KO mice showed low serum progesterone levels with an average concentration of 10.4±10.1 ng/ml, which was significantly (*P*=0.03) lower than that of WT (19.35±10.7 ng/ml) (Fig. 1L). These results strongly suggested that decreased serum progesterone levels are the primary cause for the subfertility of the *Neat1* KO mice. Notably, we frequently observed animals with normal progesterone levels in nearly half of the plug-checked *Neat1* KO mice, further supporting the aforementioned idea that *Neat1* only becomes essential in particular pregnancies.

### Prominent paraspeckle formation is observed in the corpus luteum in the ovary

**Fig. 2.**
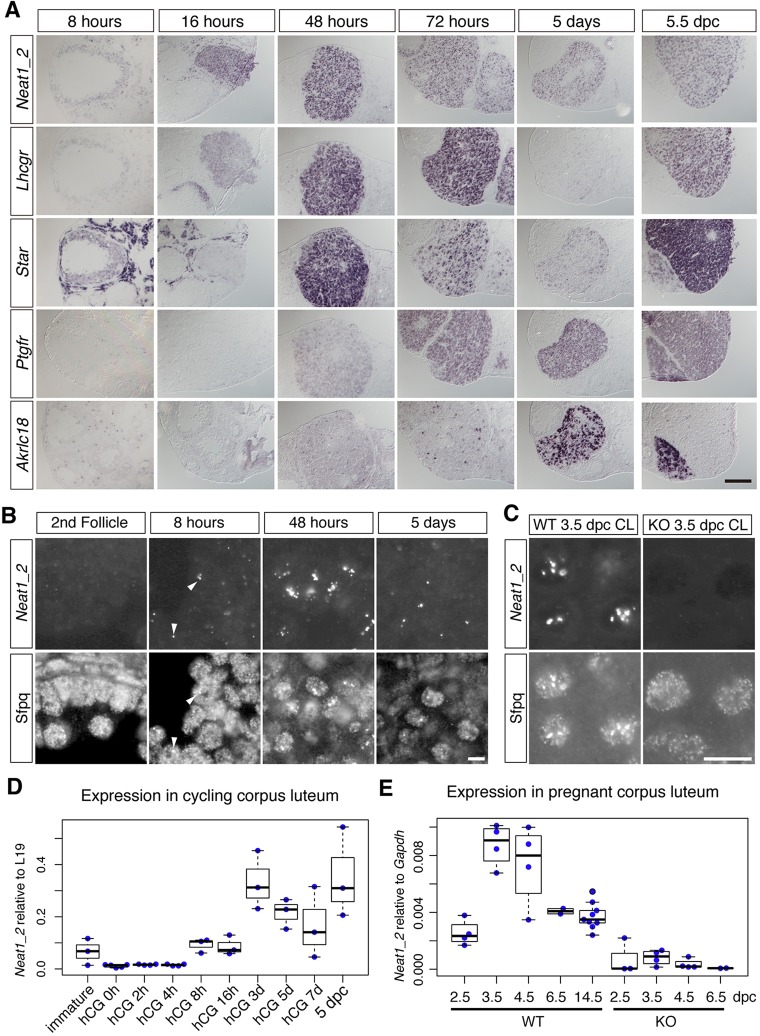
***Neat1_2* is strongly expressed in the corpus luteum and induces the formation of paraspeckles in luteal cells.** (A) *In situ* hybridization analysis of the timecourse of *Neat1_2* expression during corpus luteum development. The estrous cycle was synchronized with hCG, and the expression of *Neat1_2* and corpus luteum markers were examined in adjacent sections using *in situ* hybridization at the indicated times after hCG injection. The right column (5.5 dpc) shows the expression in the pregnant ovary. (B,C) Formation of paraspeckles during corpus luteum development. The fluorescent *in situ* hybridization signals of *Neat1_2* and immunostaining signals of the paraspeckle marker Sfpq were simultaneously detected in the cycling (B) and pregnant (C) corpus luteum. Arrowheads show accumulations of *Neat1* and Sfpq. Paraspeckle formation was not observed in the corpus luteum of pregnant *Neat1* KO mice (C). Scale bars: 100 µm (A), 10 µm (B,C). (D,E) Beeswarm boxplot of the qPCR analyses of *Neat1_2* expression in cycling (D) and pregnant (E) corpus luteum. For all boxplots, the box represents the mean, 25th and 75th percentiles; whiskers show the maximum and minimum.

Progesterone is released from the corpus luteum, which is generated from post-ovulatory follicle cells. We therefore performed detailed *in situ* hybridization analyses of the expression pattern of *Neat1_2* and luteal genes during the formation of the corpus luteum in the ovary. We first examined a timecourse of *Neat1_2* expression in the cycling corpus luteum (Fig. 2A,B) because we can synchronize the estrous cycle and control the timing of corpus luteum differentiation through the injection of hCG. The expression of *Neat1_2* was first observed in granulosa cells in the antral follicles 8 h after the injection of hCG, which coincided with the expression of *Lhcgr*, a luteinizing hormone receptor that triggers luteogenesis (Fig. 2A, 16 h). The expression of *Neat1_2* was further upregulated over the course of corpus luteum differentiation, and strong expression was observed in luteal cells 48 h after the injection of hCG (Fig. 2A, 48 h). At this time, the luteal cells also expressed *Star*, a transporter that mediates the rate-limiting step of steroidogenesis. Subsequently, *Neat1* expression was gradually downregulated in the corpus luteum coincident with the expression of the mRNA encoding the prostaglandin-F2α receptor (Ptgfr), a G-protein-coupled transmembrane receptor that induces luteolysis (Fig. 2A, 72 h). *Neat1_2* expression became even weaker in the regressing luteal cells that expressed mRNA encoding 20α-hydroxysteroid dehydrogenase (Akr1c18), an enzyme that metabolizes progesterone (Fig. 2A, 5 day). Quantitative PCR (qPCR) analysis using RNA prepared from the corpus luteum also confirmed these expression changes during the estrous cycle (Fig. 2D). We then examined the formation of paraspeckles in luteal cells during corpus luteum development (Fig. 2B). In the granulosa cells (luteal cell precursors) of early follicles, *Neat1_2* expression was not detected, and Sfpq, a marker for paraspeckles, was distributed diffusely throughout the nucleoplasm (Fig. 2B, 2nd follicle). Sfpq began to accumulate at the putative transcription sites of *Neat1_2* in the granulosa cells of the pre-ovulatory antral follicle 8 h after the injection of hCG (Fig. 2B, 8 h). Prominent enrichment of Sfpq in paraspeckles, which colocalized with *Neat1_2*, was observed 48 h after the injection of hCG (Fig. 2B, 48 h). The size and number of paraspeckles decreased during luteolysis, and the accumulation of Sfpq was observed only at the putative transcription sites of *Neat1_2* at 5 days after the injection of hCG (Fig. 2B, 5 days). We also examined the expression of *Neat1_2* and the formation of paraspeckles in the corpus luteum of pregnant mice (Fig. 2A,C). Intense *Neat1_2* signals were uniformly observed in the corpus luteum of pregnant mice (Fig. 2A, 5.5 dpc), and paraspeckles were formed in the luteal cells of pregnant mice, as revealed by the enrichment of Sfpq (Fig. 2C). As expected, paraspeckle formation was not observed in the *Neat1* KO mice, and Sfpq was diffusely distributed throughout the nuclei of the luteal cells (Fig. 2C). qPCR analyses revealed that the expression of *Neat1_2* was rapidly induced during early pregnancy (between 2.5 to 3.5 dpc), after which it gradually decreased during the middle and late pregnancy periods (Fig. 2E). As expected, the expression of *Neat1_2* was dramatically downregulated in *Neat1* KO mice, although a trace amount of the transcript was detected, especially at 3.5 dpc, when the highest expression of *Neat1_2* was observed (Fig. 2E). Taken together, *Neat1_2* was expressed throughout the entire course of corpus luteum development, with the highest expression occurring during the early phase of luteogenesis.

### Functional corpus luteum formation is impaired in *Neat1* KO mice

**Fig. 3.**
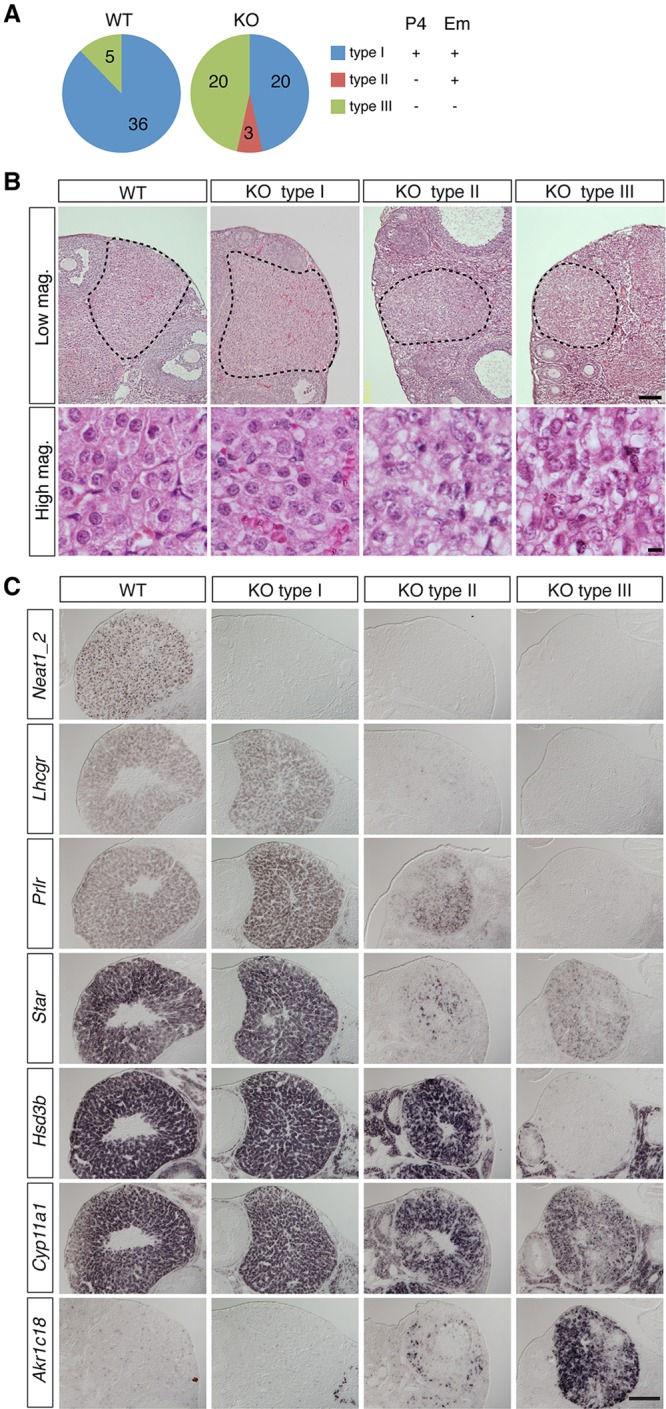
**Corpus luteum formation is impaired in *Neat1* KO mice.** (A) Pie chart of the number of WT and *Neat1* KO mice in each group (type I, II, III) categorized by the level of progesterone [P4; +, high (>10 ng/ml); −, low (<5 ng/ml)] and the presence (+) or absence (−) of implanted embryos (Em). The numbers indicate the number of animals in each group. (B) HE staining of 5.5 dpc ovaries from WT, type I, type II and type III *Neat1* KO mice. The black outlines show the corpus luteum and corpus luteum-like tissues. Note the shrunken cytoplasm of the luteal cells in the type II and type III mice. High mag., higher magnification; Low mag., lower magnification. (C) *In situ* hybridization of 5.5 dpc WT and *Neat1* KO mouse ovaries (type I, II, III) for various corpus luteum markers and *Neat1_2*. Adjacent sections were stained for each luteal gene. Scale bars: 100 μm (B, Low mag.), 10 µm (B, High mag., C).

We then examined whether formation of the corpus luteum was compromised in the absence of *Neat1*. During the course of the analyses, we noticed that naturally mated mice could be categorized into three groups according to the level of progesterone and the presence of implantation at 5.5 dpc and thereafter (Fig. 3A): type I mice possessed implanted embryos and showed increased levels of progesterone (>10 ng/ml); type II mice also possessed embryos, but their progesterone levels were low (<5 ng/ml); and type III mice showed no signs of pregnancy and low levels of progesterone. Of the WT mice, 88% belonged to type I, whereas the ratio decreased to 47% for *Neat1* KO mice. Type II mice were found only among the *Neat1* KO mice, comprising 7% (3 out of 43) of the animals. Type III mice accounted for 47% of *Neat1* KO mice, consistent with observations that nearly half of the *Neat1* KO mice stochastically failed to increase progesterone levels during pre-implantation (Fig. 3A). We then performed hematoxylin-eosin (HE) staining of histological sections prepared from 5.5 dpc ovaries of WT and *Neat1* KO mice. In WT and type I *Neat1* KO mouse ovaries, the luteal cells were readily identifiable by their pink-stained rich cytoplasm (Fig. 3B). Corpus luteum-like structures were also found in type II or type III *Neat1* KO mice (dotted line in Fig. 3B); however, the cytoplasm was largely shrunken, and vesicular spaces were observed between the cells (Fig. 3B, high magnification). We also examined the expression patterns of genes that are required for the generation (*Lhcgr*, *Prlr*), function (*Star*, *Hsd3b*, *Cyp11a1*) and regression (*Akr1c18*) of the corpus luteum in type I, II and III *Neat1* KO mice. In type I *Neat1* KO mice, the expression patterns of corpus luteum genes were almost identical to those of the WT littermates, except for the expression of *Neat1_2* (Fig. 3C). In type II *Neat1* KO mice, *Lhcgr* and *Star* expression was dramatically downregulated and *Akr1c18* expression was observed in a small population of luteal cells (Fig. 3C). In type III *Neat1* KO mice, *Hsd3b* expression was severely decreased, and strong *Akr1c18* expression was observed in the majority of the luteal cells. These observations suggest that *Neat1* is required for the expression of luteal genes in type II and type III *Neat1* KO mice.

**Fig. 4.**
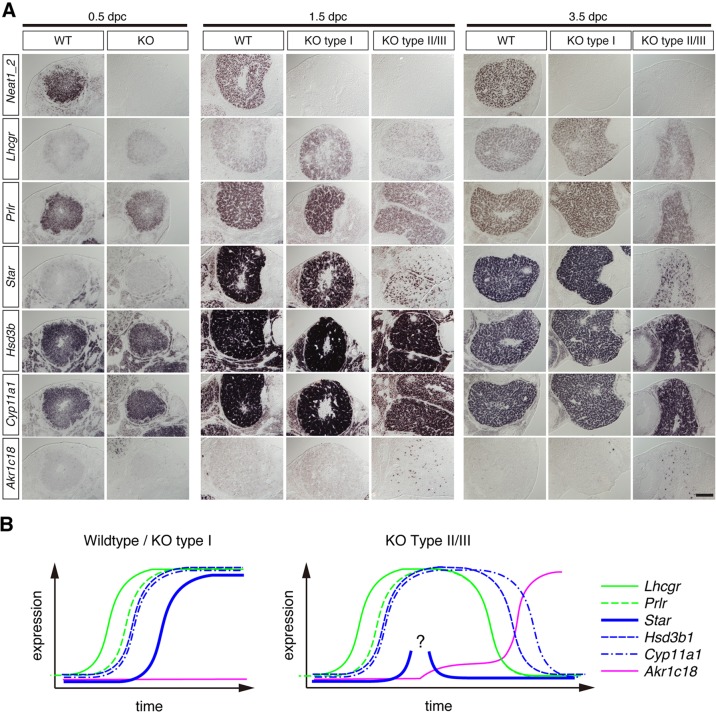
**Expression of luteal genes during the early development of the corpus luteum in *Neat1* KO mice.** (A) *In situ* hybridization of 0.5, 1.5 and 3.5 dpc WT and *Neat1* KO mouse ovaries for various corpus luteum markers and *Neat1_2*. Note that type II and type III are indistinguishable at these preimplantation stages. *Star* was not upregulated in the presumptive type II and type III *Neat1* KO mice at 1.5 and 3.5 dpc. (B) A summary of the timecourse of gene expression changes during corpus luteum development in WT and *Neat1* KO mice. The first phenotype of *Neat1* KO mice was characterized by the lack of uniform induction of *Star* in the luteal cells. Scale bar: 10 µm.

To further study the gene expression changes in the corpus luteum at the earlier preimplantation stages, we performed *in situ* hybridization analyses using ovaries obtained from WT and *Neat1* KO mice at 0.5, 1.5 and 3.5 dpc. We consistently obtained *Neat1* KO mice that showed the same expression pattern as WT mice, suggesting that they were presumptive type I *Neat1* KO mice that would have implanted embryos if the pregnancy proceeded (Fig. 4A). At 0.5 dpc, the expression pattern of the luteal genes in *Neat1* KO mice was indistinguishable from that of the WT mice as far as we tested, suggesting that the luteal development was relatively normal at this early stage (Fig. 4A, 0.5 dpc). The first sign of abnormality was the lack of uniform *Star* induction in the luteal cells of presumptive type II/III *Neat1* KO mice at 1.5 dpc (Fig. 4A, 1.5 dpc). We could not discriminate type II from type III at these pre-implantation stages, which were classified based on the presence or absence of implanted embryos. In the animals that failed to induce *Star*, a small subset of luteal cells began to express *Akr1c18* (Fig. 4A, 1.5 dpc). By contrast, the expression levels of other genes, including *Lhcgr*, *Prlr*, *Hsd3b* and *Cyp11a1*, were comparable to those of WT cells (Fig. 4A, 1.5 dpc). The impaired expression of *Star* was also observed at 3.5 dpc in the presumptive type II/III *Neat1* KO mice (Fig. 4A, 3.5 dpc). These observations suggest that expression of *Star* is initially affected in the *Neat1* KO mice, either directly or indirectly, and this is then followed by the failure of the luteal gene expression that is necessary for the proper function of the corpus luteum and increased expression of genes for luteolysis (Fig. 4B).

### Progesterone administration rescues the decreased fertility of *Neat1* KO mice

**Fig. 5.**
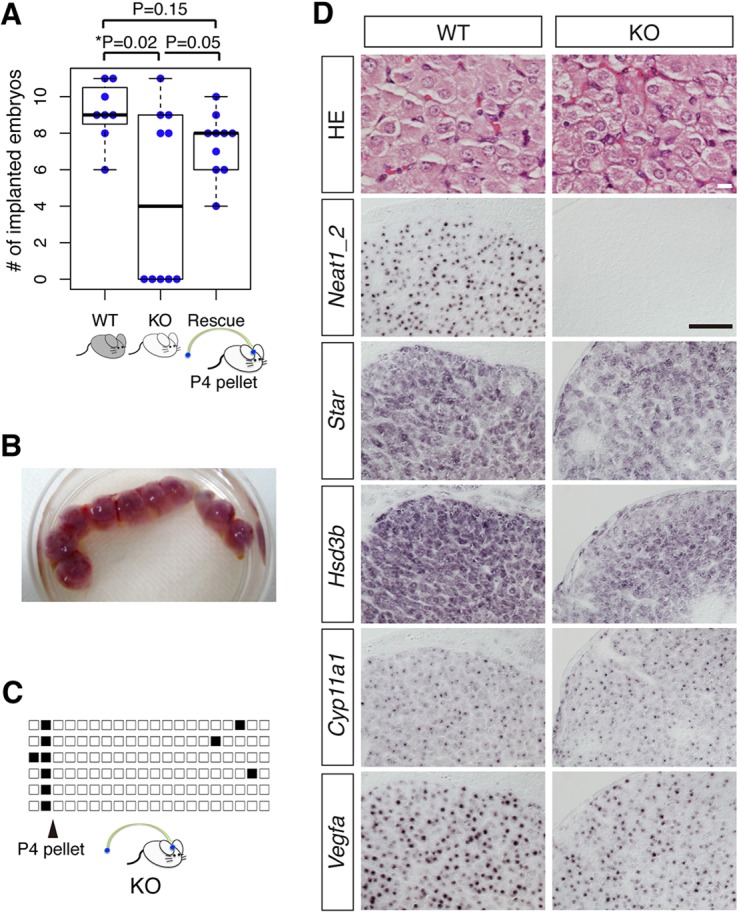
**Decreased fertility of *Neat1* KO mice is rescued by progesterone administration.** (A) Beeswarm boxplot of the number of implanted embryos in WT, *Neat1* KO and progesterone-administered *Neat1* KO (Rescue) mice at 14.5 dpc. All of the mice given progesterone became pregnant. Each blue dot represents an individual mouse. Boxes represent the mean, 25th and 75th percentiles; whiskers show the maximum and minimum. (B) The uterus of a *Neat1* KO mouse implanted with progesterone pellets at 14.5 dpc. (C) Daily record of copulation of *Neat1* KO mice transplanted with progesterone pellets. The arrowhead indicates the day of transplantation. Each black box represents the day at which plug formation was observed. Each row represents an individual animal. (D) Histological and *in situ* hybridization analysis of the corpus luteum of WT and *Neat1* KO mice transplanted with progesterone pellets. Note the slightly shrunken cytoplasm of the *Neat1* KO luteal cells. The expression of *Star*, *Hsd3b* and *Vegfa* was slightly decreased in the *Neat1* KO mice, whereas the expression of *Cyp11a1* was not affected. Scale bars: 10 µm (HE staining), 100 µm (other panels). *P* values were calculated using a two-tailed, nonequal variance *t*-test.

All of the aforementioned results suggested that the lack of progesterone synthesis was the primary cause of the decreased fertility of the *Neat1* KO mice. To further confirm this hypothesis, we subcutaneously transplanted a progesterone pellet into the plug-checked *Neat1* KO mice (Fig. 4A). Progesterone administration improved the efficiency of pregnancy, and the number of implanted embryos in the *Neat1* KO mice that were treated with progesterone was similar to that of their WT littermates (Fig. 5A,B). Progesterone administration also rescued the frequent copulation phenotypes of *Neat1* KO mice (Fig. 5C). Interestingly, we consistently observed the formation of apparently normal corpus lutea in *Neat1* KO mice transplanted with progesterone pellets, suggesting that the progesterone administration rescued luteogenesis in the presumptive type II/III mice. However, subsequent histological analysis revealed that the cytoplasm of the rescued luteal cells was shrunken in the *Neat1* KO mice (Fig. 5D), suggesting that the progesterone-rescued corpus luteum was not fully functional. Indeed, the expression of *Star* and *Hsd3b* was slightly decreased compared with that of the WT cells, whereas expression of *Cyp11a1* was not greatly affected (Fig. 5D). We also found that expression of *Vegfa*, a gene essential for corpus luteum angiogenesis (Ferrara et al., 1998), was decreased in *Neat1* KO mice transplanted with the progesterone pellet (Fig. 5D). Taken together, the post-ovulatory ability of the ovary to produce progesterone was impaired in a subset of *Neat1* KO mice, and this is the primary cause of the stochastic failure of the establishment of pregnancy.

### Apoptotic cell death does not precede dysfunction of the corpus luteum

**Fig. 6.**
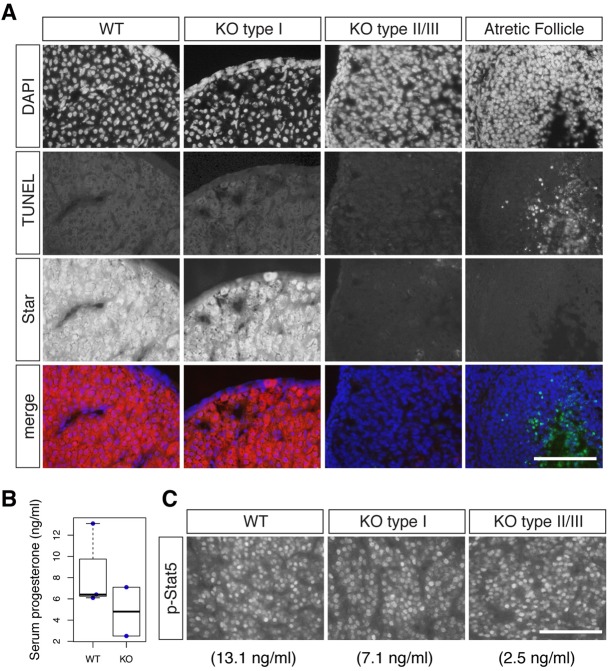
**Apoptotic cell death is not responsible for the reduction of *Star* expression.** (A) Simultaneous detection of apoptotic cell death (using TUNEL, green) and of Star (red) in the corpus luteum of WT and *Neat1* KO mice at 3.5 dpc. Note that TUNEL-positive cells are clearly found in the atretic follicles but not in the corpus luteum. DNA was counterstained with DAPI and pseudocolored in blue. (B) Beeswarm boxplot of serum progesterone levels at 1.5 dpc in WT and *Neat1* KO mice. Boxes represent the mean, 25th and 75th percentiles; whiskers show the maximum and minimum. (C) Immunohistochemical analyses of phosphorylated Stat5 in the corpus luteum. The values below indicate the serum concentration of progesterone of each individual used for the immunostaining analyses. The mice used in this study are the same as those shown in Fig. 4 (1.5 dpc). Scale bars: 100 µm.

To gain further insight into the mechanism of the corpus luteum defects in *Neat1* KO mice, we detected apoptotic cells in the corpus luteum at 3.5 dpc using a terminal deoxynucleotidyl transferase dUTP nick end labeling (TUNEL) assay. The TUNEL signals were clearly detected in the atretic follicle cells (Fig. 6A). By contrast, we did not observe prominent cell death signals in the corpus luteum of presumptive type II/III *Neat1* KO mice that lacked the expression of Star, as detected by immunostaining (Fig. 6A). These observations suggest that expression change of Star is not caused by apoptotic cell death of the luteal cells.

### The Jak-Stat pathway is normally activated in the *Neat1* KO mice

In rodents, the post-ovulatory formation of the corpus luteum in a pregnant female requires a twice daily surge of prolactin, which is initiated by cervical stimulation during copulation (Freeman et al., 1974). Prolactin signaling activates the Jak-Stat pathway in luteal cells, resulting in the accumulation of phosphorylated Stat5 in the nucleus (reviewed in Stocco et al., 2007). We thus examined the distribution of phosphorylated Stat5 in the corpus luteum at 1.5 dpc at 18.00 h, when elevated levels of the diurnal prolactin surge are observed (Barkley et al., 1978). At this stage, the average concentration of serum progesterone in WT females was 8.5 ng/ml (*n*=3), and the type I and type II/III *Neat1* KO animals showed serum progesterone concentrations of 7.1 ng/ml and 2.5 ng/ml, respectively (Fig. 6B). In all cases, phosphorylated Stat5 was observed in the nuclei of luteal cells at similar intensities (Fig. 6C), suggesting that the Jak-Stat pathway is normally activated in the corpus luteum of *Neat1* KO mice.

### *Neat1* sequesters Sfpq in paraspeckles in luteal cells

**Fig. 7.**
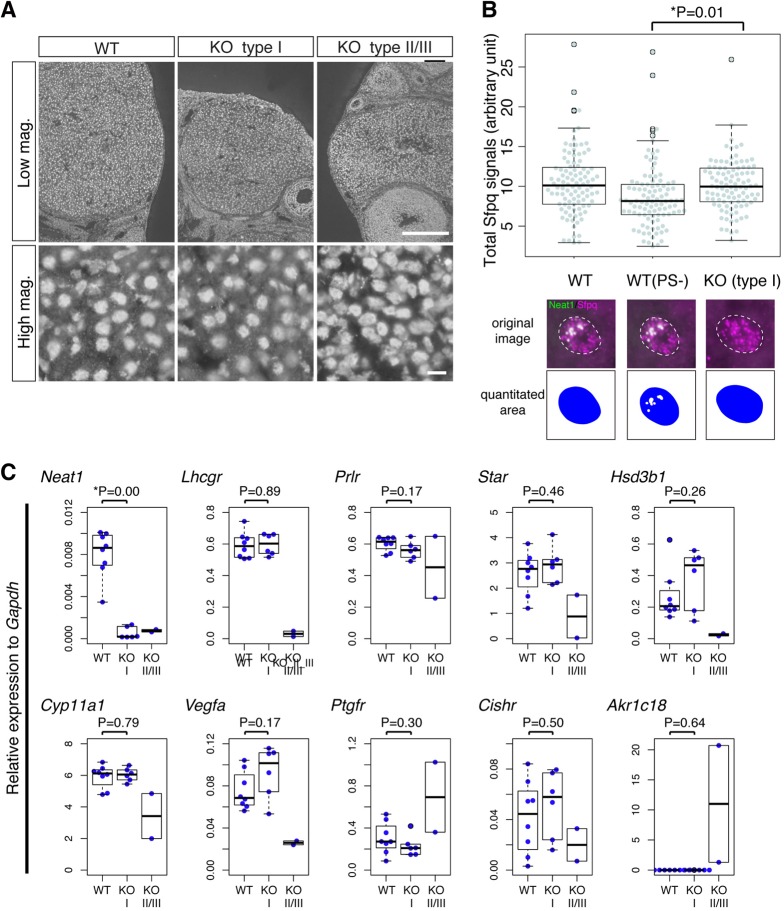
**Nucleoplasmic Sfpq was increased in the luteal cells of type I *Neat1* KO mice, but luteal gene expression was not greatly affected.** (A) Immunohistochemical detection of Sfpq in the corpus luteum of WT and *Neat1* KO mice. Scale bars: 100 µm [low magnification (Low mag.)], 10 µm [high magnification (High mag.)]. (B) Beeswarm boxplot of the quantified Sfpq signals in the nuclei of luteal cells in WT and *Neat1* KO mice. WT (PS-) indicates the Sfpq signals in the nucleoplasm outside of the paraspeckles. Paraspeckles were identified by the expression of *Neat1*, and examples of the quantified areas are shown in blue. Each dot represents an individual luteal cell that was quantified. Boxes represent the mean, 25th and 75th percentiles; whiskers show the maximum and minimum. Three pairs of WT and KO ovaries were used to perform the quantification. (C) Beeswarm boxplot of the expression of luteal genes relative to *Gapdh,* as quantified by qPCR, in WT and type I *Neat1* KO (KO) mice. Each blue dot represents an individual mouse. *P* values were calculated using a two-tailed, nonequal variance *t*-test.

Recently, it has been reported that paraspeckles regulate target gene expression by sequestering an essential paraspeckle protein, Sfpq, in nuclear bodies (Hirose et al., 2014; Imamura et al., 2014). We thus examined the expression pattern of Sfpq in the corpus luteum at 3.5 dpc. We have previously shown that Sfpq becomes diffusely localized throughout the nucleoplasm upon knockdown of *Neat1* using antisense oligonucleotides, whereas the total amount of the protein does not change (Sasaki et al., 2009). Consistently, immunohistochemical analysis revealed that Sfpq was expressed at the same level in the corpus lutea of WT and *Neat1* KO mice (Fig. 7A). We then quantified the Sfpq signal outside of the paraspeckles in the WT cells and compared this to the total Sfpq nuclear signal of the luteal cells of *Neat1* KO mice (Fig. 7B). The non-paraspeckle signal comprised 83% of the total nuclear signal, suggesting that approximately 17% of the Sfpq was retained in paraspeckles (Fig. 7B). The Sfpq signals in the nuclei of type I *Neat1* KO mice were almost identical to the total nuclear signals of the WT cells, suggesting that more Sfpq protein was available in the nucleoplasm in type I *Neat1* KO mice. We then examined whether the absence of paraspeckles and the concomitant increase in Sfpq in the nucleoplasm might alter the expression of luteal genes in the corpus luteum at 3.5-4.5 dpc by qPCR (Fig. 7C). However, we could not detect any significant changes between the WT and type I *Neat1* KO mice, which was consistent with the results obtained by *in situ* hybridization (Fig. 3C; Fig. 4A). Therefore, increased Sfpq protein levels in the nucleoplasm of the KO mice do not directly lead to changes in the expression of these genes in type I *Neat1* KO mice (Fig. 7C). By contrast, we confirmed the downregulation of luteal genes, including *Lhcgr*, *Star*, *Hsd3b1*, *Cyp11a1* and *Vegfa*, in type II/III *Neat1* KO mice by qPCR analyses (Fig. 7C).

## DISCUSSION

We demonstrated that *Neat1* is required for the formation of a functional corpus luteum under certain conditions, with nearly half of all naturally mated *Neat1* KO mice failing to establish successful pregnancy owing to low serum progesterone levels. The stochastic nature of this phenotype cannot be explained simply by differences in the genetic background because our heterozygous colony is maintained on a pure C57/Bl6 background. Moreover, the exact same mice that underwent successful deliveries irregularly failed to establish pregnancy following subsequent mating. We do not currently understand the external or internal conditions under which *Neat1* becomes indispensable for the formation of the corpus luteum. *Neat1* expression in the corpus luteum was highly variable, with a maximum change of 2.9-fold at 4.5 dpc. We thus suspect that the WT and *Neat1* KO animals were exposed to particular conditions that increased the requirement for Neat1 during the formation of the corpus luteum, resulting in a failure of the *Neat1* KO mice to form this structure.

Although the precise molecular mechanism by which *Neat1* regulates corpus luteum formation remains to be investigated, we found that expression of *Star* was the first to be affected among the luteal genes examined in a subpopulation of *Neat1* KO mice with decreased serum progesterone. *Star* regulates the rate-limiting step of steroidogenesis and is essential for the function of the corpus luteum. Expression of *Star* is controlled by multiple transcription factors that bind to its upstream promoter sequences, which include Nr5a1 (also referred to as SF-1) and Sp1 (reviewed in King and LaVoie, 2012). Interestingly, Sfpq forms a complex with Nr5a1 on the human *CYP17* promoter and suppresses Nr5a1-mediated gene activation in the adrenocortical cell line H295R (Sewer et al., 2002). Sfpq also binds to the *p450scc* promoter and inhibits the transactivator function of Sp1 in cultured porcine granulosa cells (Urban et al., 2000). These observations suggest that *Neat1* might promote the functions of Nr5a1 and Sp1 by sequestering the negative regulator Sfpq to the paraspeckles, thus facilitating the induction of *Star* during the formation of the corpus luteum. Because *Star* expression was not affected in type I *Neat1* KO mice, even though nucleoplasmic Sfpq was increased in the luteal cells, (an)other factor(s) might normally neutralize the effect of increased Sfpq in the type I ovary. Such a compensatory mechanism might stochastically fail to work, leading to the infertile phenotype observed in type II/III *Neat1* KO mice. It should also be noted that paraspeckles contain >40 RNA binding proteins (Naganuma et al., 2012), and sequestration of these proteins might regulate the expression of *Star*. Whatever the mechanism, it is essential to identify the precise environmental conditions under which *Neat1* function becomes indispensable for the formation of a functional corpus luteum.

Over the last few years, a number of studies have revealed that lncRNAs transcribed from a broad region of the mammalian genome regulate a variety of cellular processes, including the epigenetic regulation of gene expression through interactions with chromatin-modifying complexes and the control of nuclear body formation and function (Batista and Chang, 2013; Mercer and Mattick, 2013). Paradoxically, results obtained from *in vitro* studies utilizing cultured cell lines are not always consistent with the results obtained through phenotypic analyses of animal models (Nakagawa and Kageyama, 2014). In general, functional knockdowns of particular lncRNAs using antisense oligonucleotides or siRNAs lead to more dramatic phenotypic changes *in vitro* compared with animal models lacking the expression of the same lncRNAs. In this study, we showed that *Neat1* is required for the establishment of pregnancy in a subpopulation of female mice. It should be noted that the prominent phenotype of *Neat1* KO mice was observed only in the ovary, an organ that expresses extremely high levels of *Neat1_2*. In addition, the pregnancy defect was not fully penetrant. It is therefore possible that the general function of lncRNAs is cell type- and condition-specific, with these conditions being particularly represented in certain cultured cell lines. To date, there have only been a few cases in which the functions of lncRNAs have been validated in mutant animals (Grote et al., 2013; Li et al., 2013; Sauvageau et al., 2013), except for those involved in genomic imprinting (Batista and Chang, 2013; Mercer and Mattick, 2013). Further studies using animal models should provide valuable information regarding how and to what extent lncRNAs can regulate physiological processes.

## MATERIALS AND METHODS

### Animals

*Neat1* KO mice (Nakagawa et al., 2011) were extensively backcrossed to the C57BL/6 background more than ten times, and the congenic background was confirmed using 100 single nucleotide polymorphism markers that are used for the speed congenic service (Central Institute for Experimental Animals, Japan). Vasoligated mice were obtained from a local supplier (Japan SLC). For ovarian transplantation, animals were anesthetized by intraperitoneal injection of pentobarbital (50 mg/kg), and the dorsal skin area was sterilized with 70% ethanol. Small incisions were made in the skin and the dorsal peritoneal wall, and the ovary with surrounding fat was pulled out and held using curved forceps. The ovarian bursa was cut with spring scissors, and the host ovary was excised with a pair of fine forceps. A donor ovary was prepared from a WT littermate and placed into the bursa following excision of the host ovary. The donor ovaries were cut in half if they were larger than the ovary to be replaced. The peritoneal incision was closed with a single suture, and the skin was closed with three sutures. The mice were used for natural mating after 2 weeks. For sham-operated animals, the incisions were closed after the ovary was excised. For the transplantation of progesterone pellets, animals were anesthetized with pentobarbital, and the dorsal neck area was sterilized with 70% ethanol. A small incision was made in the dorsal skin with scissors, and the pellet (5 mg progesterone, 21-day release) was implanted subcutaneously. The incision was closed with a single suture. For sham operations, an incision was made and closed without pellet transplantation. To induce superovulation, 3-week-old female mice were injected with 5 I.U. of pregnant mare serum gonadotropin followed by 5 I.U. of hCG, and the eggs were recovered from the ampulla. All female mice used were younger than 20 weeks (13±3.2 weeks; mean±s.d.). All animal experiments were performed according to RIKEN animal experimental guidelines.

### *In situ* hybridization and immunostaining

*In situ* hybridization and simultaneous immunohistochemical detection were performed as described previously (Sone et al., 2007). To prepare tissue sections, dissected tissues were immersed in optimal cutting temperature compound, and the molds containing the samples were immediately frozen in a mixture of dry ice and ethanol. Sections at a thickness of 8 µm were collected on PLL-coated glass slides, fixed in 4% paraformaldehyde in Ca^2+^- and Mg^2+^-free saline buffered with HEPES (HCMF; 10 mM HEPES pH 7.4) overnight at 4°C and subsequently processed for *in situ* hybridization. For Sfpq immunostaining, the tissue sections were boiled for 20 min in HistoVT One to eliminate background signals derived from endogenous IgG. Antibodies and *in situ* probes used in this study are described in the supplementary material Tables S1 and S2. Fluorescent and differential interference contrast images were taken using an epifluorescence microscope (BX51, Olympus) equipped with a CCD camera (DP-70, Olympus) and were quantified with ImageJ software.

### qPCR analyses

To obtain RNA from the corpus luteum, luteal tissue was carefully dissected with spring scissors and fine forceps, freed from the surrounding interstitial tissues and homogenized in Trizol reagent. Total RNA (1 μg) was reverse transcribed using the ReverTra Ace qPCR RT Master Mix. Aliquots of cDNA were subjected to real-time PCR using the THUNDERBIRD(r) SYBR(r) qPCR Mix according to the manufacturer's protocol. *Gapdh* or *L19* were used as the internal normalization controls. The primers used in this study are described in supplementary material Table S3.

### TUNEL staining

Apoptotic cell death was detected using an In Situ Cell Death Detection Kit (Fluorescein) according to the manufacturer's instructions. Briefly, freshly frozen tissue sections were fixed in 4% paraformaldehyde in HCMF for 1 h at room temperature, washed in HCMF and permeabilized in 100% methanol at −20°C for 5 min. After rehydration with HCMF, the sections were equilibrated with 1× TdT buffer and subsequently incubated with the labeling mix for 1 h at 37°C. After washing with TBS, the sections were incubated with an anti-Star antibody for double staining.

## Supplementary Material

Supplementary Material
