# Supplementary material for: The lncRNA Neat1 is required for corpus luteum formation and the establishment of pregnancy in a subpopulation of mice
Source: Development. 2014 Dec;141(23):4618–27. doi: 10.1242/dev.110544 (PMC4302932; doi:10.1242/dev.110544)
Supplement: Supplementary Material [file supp_141_23_4618__index.html]

Supplementary Material 

# The lncRNA *Neat1* is required for corpus luteum formation and the establishment of pregnancy in a subpopulation of mice

## DEV110544 Supplementary Material

**Files in this Data Supplement:**

- Supplementary Material
