# Supplementary material for: The lncRNA Neat1 is required for corpus luteum formation and the establishment of pregnancy in a subpopulation of mice
Source: Development. 2014 Dec;141(23):4618–27. doi: 10.1242/dev.110544 (PMC4302932; doi:10.1242/dev.110544)
Supplement: Supplementary Material [file supp_dev.110544_DEV110544supp.pdf]

Table S1. Antibodies

Anti-Sfpq antibody [B92]	ab11825, Abcam
Anti-Sfpq antibody	A301-321A, Bethyl Laboratory
Anti-Star antibody [D10H12]	#8400, Cell Signaling Technology
Phospho-Stat5 (Tyr694) (C11C5) Rabbit mA	#9359P, Cell Signaling Technology
Mouse Anti-Digoxigenin Monoclonal Antibody	1333062, Roche
Rabbit polyclonal anti-FITC antibody	ab73831, Abcam
Cy3-conjugated goat anti-mouse antibody	AP124C, Millipore
Alexa Fluor 488-conjugated anti-rabbit antibody	A11029, Invitrogen

Table S2. Probes used for in situ hybridization

Neat1_1	Descibed in Nakagawa et al. (2011)
Neat1_2	Descibed in Nakagawa et al. (2011)
Lhcgr	RIKEN Fantom Clone E330016L12
Prlr	RIKEN Fantom Clone D630022K08
Star	RIKEN Fantom Clone 8030467N13
Hsd3b	RIKEN Fantom Clone I920176L22
Cyp11a1	RIKEN Fantom Clone 6030456M03
Akr1c18	RIKEN Fantom Clone E430003M17
Vegfa	RIKEN Fantom Clone 6330442K08

Table S3. qPCR primers (annealing 60ºC)

Gene	Forward	Reverse
Gapdh	cctcgtcccgtagacaaaatg	tctccactttgccactgcaa
L19	ggcatagggaagaggaagg	ggatgtgctccatgaggatgc
Neat1_2	gctctgggaccttcgtgactct	ctgccttggcttggaaatgtaa
Lhcgr	agatgcacagtggcaccttc	gtaggatgacgtggcgatga
Prlr	ggcatagggaagaggaagg	ggatgtgctccatgaggatgc
Star	caggaaggctggaagaagga	ggctggtctaccaccacctc
Hsd3b1	ttcagccaccaccatctcag	tggtctgtccttcccagtga
Cyp11a1	ccaaggatgcgtcgatactc	cggtctttcttccaggcatc
Vegfa	aaaacacagactcgcgttgc	ctttccggtgagaggtctgg
Ptgfr	cactcagtggctcaggatgc	acactggctgcttggaactg
Akr1c18	gccaggccattctaagcaag	gctgggtctgaccaactctg
Cishr	gccgaactccgactctcgag	ccgggcagctccagggactg
